# Interpreter-mediated diabetes consultations: a qualitative analysis of physician communication practices

**DOI:** 10.1186/1471-2296-14-163

**Published:** 2013-10-24

**Authors:** Patricia Hudelson, Melissa Dominicé Dao, Noelle Junod Perron, Alexander Bischoff

**Affiliations:** 1 Department of Community Medicine, Primary Care and Emergency Medicine, University Hospitals of Geneva, Geneva, Switzerland

**Keywords:** Diabetes, Interpreters, Communication

## Abstract

**Background:**

Patient-provider communication, in particular physicians’ ability to listen to their patients, and support them in making difficult lifestyle changes, is an essential component of effective diabetes care. Clinical communication around diabetes can be especially challenging when language barriers are present, and may contribute to poor diabetes management and outcomes. Clinicians need to be aware of and address potential communication difficulties associated with interpreter-mediated consultations. The purpose of our study was to explore how physicians communicate in interpreter-mediated consultations with diabetic patients, and how their communication behaviors may impact diabetes communication and care.

**Method:**

We analyzed transcripts from 8 audio recorded, outpatient consultations at the Basel University Hospital general medicine outpatient clinic involving Turkish-speaking patients, German-speaking physicians, and Turkish-German interpreters (both community interpreters and family members).

**Results:**

Clinicians used closed questions when asking about symptoms and glucose control. When providing information and explanation, they spoke in long and complex speech turns. They often directed their speech to interpreters or became sidetracked by family members’ questions or requests for information. Patients’ participation in the consultation was minimal, and limited to brief answers to clinicians’ questions.

**Conclusions:**

Clinicians need to be aware of common pitfalls that diminish patient-centeredness during interpreter-mediated consultations, and learn strategies to avoid them. Attention to established guidelines on triadic communication is recommended, as is hands-on training with interpreters.

## Background

### Diabetes and the importance of patient-provider communication

Diabetes is an enormous public health challenge and a major cause of morbidity, mortality and health care costs [1]. In Switzerland there are currently more than 250’000 diabetics, and the World Health Organization estimates that this number will grow to 300'000 by 2025 [2].

Although there is strong evidence that good diabetes control leads to improved clinical outcomes, many people with diabetes continue to have inadequate glycemic and blood pressure control and elevated low-density lipoprotein levels [3]. Poor adherence to treatment has been identified as an important factor contributing to poor diabetes control and outcomes, and is a complex problem. Common barriers to treatment adherence include patients’ lack of understanding of diabetes and its complications, low motivation to make lifestyle changes and social and financial problems [4].

Physicians face a significant challenge in helping patients manage their diabetes and improve their health and quality of life [5]. Patient-provider communication--specifically physicians’ ability to elicit patients’ concerns, explore contextual factors and patient’s beliefs about health and illness and support them in making difficult lifestyle changes--is essential to effective diabetes care [6-8], and a number of studies have shown that enhanced patient-provider communication and shared decision-making can lead to improved adherence to treatment plans and improved health outcomes [9].

### The challenges of interpreter-mediated clinical communication

Clinical communication around diabetes is especially challenging in contexts of cultural and linguistic diversity [10]. In multicultural contexts, patients and physicians often differ in their understanding of illness, their values and preferences regarding treatment, expectations of the clinical encounter, and communication styles [11-16]. These differences can lead to misunderstandings [17-19]. Understanding patients’ perspectives and addressing cultural barriers to care are important for ensuring effective communication and diabetes control [20-22].

When patients and physicians do not speak a common language, interpreters are often perceived as the solution for bridging patient-provider linguistic and cultural differences [23]. A number of studies indicate that use of trained, professional interpreters is associated with improved clinical care [24]. In one study, the use of professional interpreters for non-Anglophone patients contributed to ensuring the same quality of care as that provided to English-speaking patients [25].

Many studies have examined the effect of interpreter skills and practices on the quality of communication and care [23,24,26-34]. However, the quality of interpreter mediated clinical communication depends on the skills and practices of both the interpreter and the clinician, and on their effective collaboration. Most guidelines recommend that physicians speak directly to patients, speak in short phrases, avoid jargon, ask one question at a time, and pause frequently to allow interpretation [35-39]. These recommendations are meant to create optimal conditions for interpreters so that they may accurately translate all that is said. Unfortunately, in many contexts practices remain suboptimal. Not all physicians receive training in how to work with an interpreter, and many may overestimate their skills [40-42]. In a study in Geneva, we found that while physicians and medical students considered themselves highly skilled, most were unable to name basic elements of effective collaboration with an interpreter [42].

Clinicians frequently rely on untrained interpreters (family members and untrained bilingual hospital staff) despite the fact that such interpreters have been associated with clinically relevant interpretation errors, non-neutrality and confidentiality problems [23,43]. Untrained interpreters may have difficulty understanding and accurately interpreting key concepts related to diabetes and diabetes control [44], and studies have shown that unbeknownst to physicians, interpreters often actively influence the medical encounter [27,30,45-47]. Clinicians untrained in how to work with interpreters may not be aware of these shortcomings nor have skills to manage the specific communication difficulties that arise with untrained interpreters.

The interpreter-mediated medical encounter is a complex interaction between providers, interpreters and patients, but only a few studies have specifically explored how provider communication patterns affect patients’ participation in interpreter-mediated consultations ( [48,49]. The aim of our study was to examine physicians’ practices in light of current “best practice” guidelines.

### Objectives

We explored physicians’ communication behaviors in interpreter-mediated consultations with Turkish immigrant diabetic patients, with the aim of identifying difficulties and areas for improvement.

## Methods

### Study site

The study was conducted at the general medicine outpatient clinic (Medizinische Universitäts poliklinik, or MUP), which is part of the University Hospital Basel (UHBS). The UHBS is a teaching hospital that provides primary, secondary, and tertiary care for a population of approximately 200,000 people. The general medicine outpatient clinic (Medizinische Universitäts poliklinik, or MUP) provides about 17,000 internal medicine consultations each year. The medical staff consists of 11 residents in internal medicine, most of whom have completed more than four years of postgraduate clinical training, and 5 attending physicians who supervise them [50]. Patients at the MUP reflect diverse socioeconomic, cultural and linguistic backgrounds. To assist communication with non-German speaking patients, a community interpreter service run by the Swiss Interchurch Aid agency (Linguadukt) is available to MUP staff.

Community interpreting has been active in Switzerland since the 1990s, and there are currently 21 community interpreting agencies in the country. Formal training of community interpreters and training of clinicians to work with community interpreters is a relatively recent development. Community interpreting training standards were developed in 2002, a certificate in Community Interpreting was first offered in 2004, and a nationally recognized certification exam was created in 2009 [51]. However, many community interpreters have yet to benefit from these training and certification opportunities. Training of clinicians to work effectively with community interpreters is even more recent, and not yet systematically offered to nor obligatory for physicians and other health care professionals. At the time of this study, no Linguadukt interpreters had yet been certified (although they received some minimal training/orientation when hired) and MUP staff had received no formal training in how to work with interpreters.

### Data collection

During August-September 2005, scheduled consultations at the MUP involving a Turkish-speaking diabetic patient in need of an interpreter were noted. We focused on Turkish-speaking patients because they are an important immigrant population in Basel [52] and among whom diabetes is a common health problem [53].

The research assistant was informed by the head physician when there was to be a consultation involving a Turkish-speaking diabetic patient and an interpreter (professional or ad-hoc). At the beginning of the consultation, the research assistant explained the study objectives, invited all 3 parties to participate (physician, patient, interpreter) and provided informed consent forms (in Turkish for patients). If all three parties accepted to participate, the research assistant remained in the consultation room and recorded the interview. Ethical clearance for the study was obtained from the EKBB (Ethikkomission Beider Basel).

All consultations were fully transcribed at the time of data collection. The research assistant transcribed all German utterances, and a Turkish-speaking university student transcribed the Turkish utterances. The Turkish utterances were then translated to German by a bilingual research assistant.

The analysis team consisted of one bilingual German/English researcher (AB) and 3 bilingual English/French researchers. To allow for analysis by non-Germanophone researchers, and to facilitate subsequent publication of results in English, we had the German transcripts translated into English by a professional translator, and double checked by AB. During analysis, AB referred to the German transcripts, while the other researchers worked from the English transcripts.

Because we were concerned about potential distortions of the Turkish portions due to double translation (Turkish to German to English), we had one full transcript analysed by an independent, trilingual (Turkish-German-English) professional translator. This translator found no errors and considered the translation to be very accurate. During analysis, on the rare occasions where doubts or questions arose about the Turkish utterances, a local Turkish community interpreter (not involved in the study) was consulted.

### Analysis

Researchers initially read each transcript independently with the aim of identifying key passages and themes that illustrated physician speech patterns, interpreter roles and patient participation. Researchers then met to compare and discuss their general impressions of the transcripts and develop a list of codes that were then applied to all transcripts by PH using the qualitative data analysis software MAXQDA2007®. Coding and analysis were then cross-checked by the other members of the research team. When presenting quotes from the consultations, we have used italics to indicate where Turkish was spoken and normal text when German was spoken.

## Results

Eight interpreter-mediated general medicine consultations were audio recorded at the MUP. There were no refusals to participate. Consultations involved 8 clinicians (5 medical doctors, 1 nurse, 1 dietician and 1 diabetic educator), 2 professional interpreters and 7 patients, and reflected three configurations: 4 consultations involved a family member as interpreter, 2 consultations involved both a family member and professional interpreter, and 4 consultations involved only a professional interpreter. Table 1 provides a summary description of each of the 8 consultations.

**Table 1:** Overview of consultation characteristics

**Interview ID**		**Clinician**	**Who interprets**	**Patient**	**Reason for consultation**	**Patient**	**Clinician**’**s speech style**	**Interpreter**’**s speech style**
Professional interpreter, no family present	A	Female doctor	Female professional interpreter D1	Male patient, 60 yrs	Diabetes check-up, and chest/arm pain. Much of the discussion centers on understanding the patient’s chest and arm pain symptoms.	Patient speaks no German	Clinician always addresses interpreter	The interpreter frequently asks the patient additional questions or for clarification before interpreting back to the clinician, in an attempt to provide the doctor with coherent answers to her questions.
	D	Female doctor (same as E)	Female professional interpreter D1	Female patient, 50 yrs	Diabetes check-up. The discussion centers on when and how she measures her glucose, and on adjusting her insulin.	Patient speaks a little German	Doctor most addresses the patient directly.	Patient and doctor speak to each other in German; the interpreter only intervenes occasionally to interpret when the patient switched to Turkish or if the patient seemed not to understand the doctor.
	E	Female doctor (same as D)	Female professional interpreter D1	Female patient, 33 yrs	Diabetes check-up. The discussion centers on her glucose measurements and how to calculate and adjust her insulin.	Patient speaks a little German	Doctor mostly addresses patient directly, and is quite empathetic and encouraging. She speaks in lengthy turns, with a lot of numbers.	Patient appears to understand at least some German because the interpreter doesn’t bother to translate everything, and sometimes the patient answers (in Turkish) the doctor’s question before it has been translated. The interpreter does a lot of paraphrasing of doctor’s speech.
	F	Female doctor, and female nurse diabetic educator	Female professional interpreter D2	Female patient, 58 yrs	Diabetes check-up. The discussion centers on review of her glucose measurements, how to adjust her insulin, and the importance of seeing the dietary counsellor.	Patient speaks no German	Clinician mostly addresses interpreter	Interpreter often uses first person translation for the patient and direct or reported speech for the clinicians.
							The clinician speaks in lengthy turns	This is the only consultation where the interpreter intervenes to suggest that the patient may not understand the doctor’s explanations.
Family as interpreter	H	Female doctor	Adult son	Husband and wife together, 70 yrs	Diabetes check-up. The doctor sees husband and wife together to go over various lab results.	Husband and wife both speak a little German	Doctor almost exclusively addresses patients directly, but is frustrated by the son who doesn’t translate much.	Son answers for parents, rarely translates, and asks his own questions.
	G	Male doctor	Adult daughter	Female patient, 60 yrs	Results from lung x-ray; diabetes check-up. The discussion centers on the patient’s x-ray results, as well as her diabetes and blood pressure management.	Patient speaks a little German	Doctor addresses patient directly, but is drawn into conversation with the daughter.	Daughter answers for mother, translates only when she cannot answer the doctor’s question herself. She also asks her own questions.
Professional interpreter and family	C	Female nurse/diabetic educator	Female professional interpreter D1	Female patient, 40 yrs, accompanied by her adult daughter	Gestational diabetes. The discussion centers on the importance of the patient following her diet plan more closely in order to determine whether or not she needs insulin.	Patient speaks no German	The clinician starts by addressing the interpreter, but when the daughter jumps in to answer a question for her mother, the clinician then talks only to the daughter.	At first the interpreter attempts to translate for the patient what is being said between the clinician and daughter, but is not given the time and eventually becomes silent while clinician and daughter speak for the rest of the consultation.
	B	Female dietician	Female professional interpreter D1	Female patient, 40 yrs, accompanied by her adult daughter (**same as in B**)	Gestational diabetes. The discussion focuses on her dietary plan.	Patient speaks no German	Clinician speaks directly to the patient, never the interpreter	The interpreter tends to summarize the clinician’s lengthy turns. The daughter says nothing during the consultation, and only responds to a question about a next appointment.
							The clinician speaks in lengthy turns	

Several communication patterns of clinicians were identified that can act to limit patient participation. While some are specific to working with interpreters, others are potentially problematic even where there is no language barrier. The examples provided point to the importance of general patient-provider communication techniques as well as specific attention to the challenges of interpreter-mediated communication.

### Use of closed questions and directive statements

The use of patient-centered communication techniques such as open-ended questions, empathy, and engagement of patients in discussion is thought to be an important aspect of chronic disease management [10,54-57].

Clinicians in our study used mainly closed questions (98/103 questions), made directive utterances (“Test your blood glucose on an empty stomach”) and made statements about objective reality (“your glucose is too high”). They rarely elicited patients’ feelings, opinions or difficulties, and there was little collaborative problem-solving discussion.

Not surprisingly, patients tended to provide mainly short (often just one or two word) answers to clinicians’ questions, and rarely asked questions of their own or spontaneously offered information. Only 4 patients (A, C, D, E) made a total of 8 spontaneous requests for advice, information or clarification regarding their health (for example, “Where does my kidney problem come from?” “Is my glucose too high now?”). Of these, only 4 were translated back to and answered by the clinician. The other 4 questions were either ignored or answered directly by the interpreter.

### Speaking in lengthy turns

To facilitate the work of interpreters and increase the likelihood of an accurate and complete translation, clinicians should speak in relatively short turns (one or two sentences or ideas at a time), and ask only one question at a time.

In our study, clinicians spoke in short turns when asking questions about patients’ symptoms (“Where does it hurt?” “When did it start?”), but spoke in long turns when providing explanations or instructions to patients (we defined lengthy as 4 or more statements or questions at a time). This practice was especially frequent in consultations D and E, where patients understood a little German, despite the fact that their German was not good enough to forego interpretation. This may indicate that clinicians overestimated the patient’s language skills, or that they were simply unaware of the difficulty of the interpreter’s task. At one point in Consultation E, the doctor spoke 25 sentences before pausing. Interpreters in our study never asked clinicians to pause, but rather summarized the speaker’s words as best they could, often omitting or modifying the content, as in the example below:

Dr: So that in the morning/ because exactly, if you have twenty-seven, and you inject ten, then/then it, ten would correct twenty, wouldn’t it? Then ((2s)) yes… Exactly/ that’s it, seven. But in the evening sometimes people are more sensitive to insulin. Then/ especially when their glucose drops quickly, then exactly, it also causes the feeling of hunger. And when you then eat, your levels start to swing, hm? Okay. We’ll make it like that in the evening from now on..[P: Mm] And you go to the Aeschengraben [Mm] and try and get your personal life a bit more under control again. That will surely require time. When your personal life is a bit more peaceful, the eating will also calm down, and then we can start working, hm? And you’ve got the idea, so it’s no great disaster, hm?

P: Mm.

I: *Well*, *your glucose is not bad*, *she says*. *Only you should do what we*’*ve talked about*, *she says*. *Apart from that*, *she says go to um the Aeschengraben*. *Try to get certain things in your personal life in order*. *She says if these get better*, *things will*… [*P*:*Mm*] *She says you should sort out all the things that need sorting out*. *She says when you do it in that way*, *then I think*, *she says*, *that the glucose will sort itself out*.

### Not checking for patients’ understanding

It has generally been recommended that physicians’ check for patients’ understanding of medical information by asking them to repeat back what they have understood, rather than simply asking “Do you understand?” [58,59] In our study, no clinicians used the check-back method. In Consultation A the patient asks the doctor why he should keep his kidney if it doesn’t work anymore. After a lengthy explanation from the doctor about how kidneys function, the doctor asks “So, reasonably well understood?”, to which the interpreter responds “Mm”, but the question is not translated back to the patient, and doctor goes on with his discussion of dialysis. In Consultation H the doctor tells the patient to stop taking one of her medicines, and then asks “Have you understood?” The patient’s son replies that his mother has understood, but the patient says nothing and the doctor continues talking.

### Directing speech to interpreters

When working with interpreters, clinicians should direct their speech to the patient. This is intended to simplify the interpreter’s task by eliminating the extra mental task of reformulation, such as when the doctor says “Ask him if he has a headache” and the interpreter must reformulate this to “Do you have a headache” or even “He wants to know if you have a headache”. Speaking directly to the patient can also facilitate the patient/provider relationship through eye contact and non verbal interaction.

In our study, only the dietary counsellor in consultation C consistently addressed her patient despite the fact that the patient did not speak any German. Clinicians in the other consultations spoke directly to patients who understood a little German (consultations D, E, G, and H), but to interpreters when patients spoke no German at all (consultations A, B, F).

Consultation B:

Dr: Mm. Um would she like to have a look what such a pen looks like already, or would she rather simply tackle that issue when it’s necessary?

By addressing their questions to interpreters, clinicians essentially put them in the role of “expert”, which sometimes led to the interpreter to either answer for the patient or take initiative to reformulate questions or dig for more details. By allowing the interpreter to have a side conversation with the patient, the clinician is excluded from the interaction.

Consultation A:

Dr: Does he feel it’s got stronger or worse or more frequent in the last while?

I: *Have the pains got worse with time*?

P: *Well*, *in the last while*…*the pains*/*since night*, *I*’*m in pain all the time*) [*I*:*Mm*] *I don*’*t know why*, *was it after eating or why*…

I: *Where does it hurt then*?

P: *Here*, *here it hurts* [*I*:*Mm*] *In bed*, [*I*:*MmMm*] *I couldn*’*t sleep*, *got up and had a shower* ((*1s*)) *I went back to sleep very slowly* ((*2s*)) *And then while I was asleep I had bladder pressure and wake up again*.

I: *Mm*. *Mm*. *So you were in more pain in the last while*? *Has the pain got worse in the last*?

P: *I went to this swimming pool*. *At the swimming pool it didn*’*t hurt* [*I*:*Mm Mm*] *Um*, *I went home*, *at home it started again*.

I: Okay. So he couldn’t give a direct answer to my question. I’ve tried several times [Dr: Mm]…

### Allowing family members to sidetrack the conversation

Family members can be a source of support and encouragement for patients and can sometimes provide additional information or help patients to remember details [60]. However, they can also impede patient/provider communication by failing to interpret all that is said, by interpreting incorrectly, by answering the clinician’s questions directly in place of the patient, and by engaging the clinician and/or patient in side conversations [61,62].

In consultations G and H, the patients’ adult child acted as interpreter. In both these consultations the clinicians were sidetracked by family members’ questions and comments, which led to the exclusion of patients from the interaction.

In consultation G, despite the clinician’s repeated attempts to speak directly to the patient, the conversation is sidetracked several times by the son’s requests for explanation. In the excerpt below, the doctor is trying to explain to the patient that he will send her again for a Diabetic Education session, but rather than translating, the son asks his mother what diabetes is, and when he doesn’t get an answer from his mother he asks the same question to the doctor.

Consultation G

Dr: Well Mrs Yılmaz. Then we will have to start all over again. Then I will send you to Diabetic Education again.

Patient: ((incomprehensible, 1.5s))

Son: *What is diabetes*?

Patient: Y*ou haven*’*t understood anything*.

Dr: Exactly, please translate this for (her) now.

Son: What is diabetes? I don’t know myself.

The son draws the doctor into conversation numerous times during the consultation, and the frustrated doctor reminds him five times to interpret for his parents, to no avail.

In Consultation H, the doctor is quite persistent in speaking directly to the patient, but the daughter repeatedly answers for her mother and often fails to translate, as in the example below. Although the doctor receives an answer to his question, the patient is excluded from the interaction.

Dr: Then uum you must go to Diabetic Education again. That’s important, hm? I’ll register you again with Diabetic Education. You’ll just have to go there again so that they can discuss it with you. And I’ll look ((incomprehensible, 0.5s)).

I: She did it for a year…she doesn’t do exactly the same at home… I translate it for her and everything but she doesn’t do the same at home…that’s the problem.

There was less sidetracking in consultations B and C, where both the patient’s daughter and a professional interpreter were present. In consultation C, the dietary counsellor speaks directly to the patient at all times, and the interpreter translates. The daughter only speaks when the clinician asks her a direct question at the end of the consultation about a next appointment for her mother. In consultation B, however, the daughter speaks much more. The clinician asks her a question near the beginning of the consultation (“Do you write it [glucose values] down each time?”). The daughter answers the question, and from this point on the clinician shifts her attention to the daughter, never speaking directly to the patient again. At first the interpreter attempts to translate the exchange for the patient, but as there are no pauses in the conversation she finally gives up and is silent for the rest of the consultation, as is the patient.

## Discussion

We explored how clinicians communicate with diabetic patients in interpreter-mediated consultations. We found that the essential elements of both patient-centered communication and provider/interpreter collaboration were often lacking [63]. Communication tended to be one-sided and clinician-centered, with clinicians doing most of the talking, and controlling the conversation by using closed questions and directive speech. When family members translated, clinicians had difficulty managing the flow of conversation and staying focused on patients. When working with professional interpreters, clinicians directed their speech towards interpreters, spoke in long turns and asked several questions at once. These speech patterns acted to limit patient participation in the conversation.

Our study was small and exploratory, involving only 8 clinicians and 2 interpreters. Had we broadened our sample to include other clinicians and clinical contexts, we might have identified additional practices. We were also unable to explore how individual clinicians’ communication styles vary according to the presence/absence and type of interpreter (family member vs. professional). For example, we don’t know whether clinicians in our study use more patient-centered communication techniques with their German-speaking patients. A Dutch study found, for example, that doctors were more verbally dominant with immigrant patients and showed more empathy and involvement with Dutch patients [64].

Despite the limited scope of our study, our findings resonate with findings elsewhere. For example, Seale and colleagues [49] found that speech patterns of both patients and clinicians were different in interpreter-mediated diabetes consultations as compared with English-only consultations. In triadic consultations patients spoke less, asked fewer questions, and talked less about their illness ideas and life circumstances. They also found that providers rarely spoke directly to patients, explored patients’ lifeworld less often and used humor less frequently. Kokanovic and Manderson [10] found that triadic consultations were often limited to clinical assessment and brief discussions about patients’ physical health, with little opportunity to discuss the social and personal impact of diabetes. Rivadineyra et al. found that patients in interpreter-mediated consultations made fewer comments, were less likely to receive facilitation from their physicians and were more likely to have their comments ignored than English-speaking patients [48]. These findings suggest that communicating effectively through an interpreter is not intuitive, and requires attention to the pitfalls of triadic communication. Clinicians need formal training in order to collaborate effectively with an interpreter and ensure patient-centered communication in interpreter-mediated consultations [40,42].

## Conclusions

Effective communication is the key to patient-centered care [65]. A lack of expertise in applying clinical communication skills in interpreter-mediated consultations can lead to suboptimal communication and care. Systematic training of clinicians and attention to best practice guidelines are needed to reduce the social distance of interpreted consultations and encourage more patient involvement, which is essential to effective self-management of diabetes [49]. Training efforts should challenge the assumption that working with an interpreter is intuitive [42], raise clinicians’ awareness of the interpreter’s role and requirements, and provide practical strategies for preventing and managing common pitfalls of interpreted consultations.

## Competing interests

The authors declare that they have no competing interests.

## Authors' contributions

All authors were involved in defining the research question and developing an initial analysis plan. PH analyzed and coded the transcripts, with feedback from NJP, MDD and AB. PH drafted the manuscript. All authors critically reviewed the manuscript. All authors read and approved the final manuscript.

## Pre-publication history

The pre-publication history for this paper can be accessed here:

http://www.biomedcentral.com/1471-2296/14/163/prepub
